# Real-Time Detection of Slug Velocity in Microchannels

**DOI:** 10.3390/mi11030241

**Published:** 2020-02-26

**Authors:** Salvina Gagliano, Giovanna Stella, Maide Bucolo

**Affiliations:** Department of Electrical, Electronics and Computer Engineering, University of Catania, v.le A. Doria 6, 95129 Catania, Italy; salvina.gagliano@unict.it (S.G.); giovanna.stella@phd.unict.it (G.S.)

**Keywords:** experimental study, optical signals monitoring, air–water flows, slug velocity, slug frequency

## Abstract

Microfluidics processes play a central role in the design of portable devices for biological and chemical samples analysis. The bottleneck in this technological evolution is the lack of low cost detection systems and control strategies easily adaptable in different operative conditions, able to guarantee the processes reproducibility and reliability, and suitable for on-chip applications. In this work, a methodology for velocity detection of two-phase flow is presented in microchannels. The approach presented is based on a low-cost optical signals monitoring setup. The slug flow generated by the interaction of two immiscible fluids {air and water} in two microchannels was investigated. To verify the reliability of the detection systems, the flow nonlinearity was enhanced by using curved geometries and microchannel diameter greater than 100 μm. The optical signals were analyzed by using an approach in a time domain, to extract the slug velocity, and one in the frequency domain, to compute the slug frequency. It was possible to distinguish the water and air slugs velocity and frequency. A relation between these two parameters was also numerically established. The results obtained represent an important step in the design of non-invasive, low-cost portable systems for micro-flow analysis, in order to prove that the developed methodology was implemented to realize a platform, easy to be integrated in a System-on-a-Chip, for the real-time slug flow velocity detection. The platform performances were successfully validated in different operative conditions.

## 1. Introduction

Nowadays, the hydrodynamics of two-phase slug flows in microchannels [1] play an important role in the micro-nano technology, enabling the design for lab-on-chip devices in the bio-medical field as well as in chemical processes [2,3]. In this context, an open issue is that of developing detection systems, models, and control strategies easily adaptable in different operative conditions, are low-cost, able to guarantee the processes reproducibility and reliability, and suitable for on-chip applications [4,5].

The results presented in literature are strictly related to specific experimental conditions, so far from a well-established framework that can drive to the flow control. Recently, some case studies using System-on-a-Chip (SoC) predictive control strategies have been presented in literature [5,6]. The SoC offers a high control level and modularity, but its functionalities are strongly dependent on the integrated control logic and the knowledge of the process model. In this complex scenario, in which the properties of the fluids, the input flow conditions, the channel geometry and the material surface properties can strongly affect the flow dynamics, the need to develop detection systems, models, and control strategies completely independent of any constraints related to the experimental conditions represents the bottleneck for widespread diffusion that SoC uses in microfluidics applications.

In two-phase slug flows, two immiscible fluids, one dispersed in the other, are circulating in the same microsystem (for instance gas–liquid, immiscible liquid–liquid, or liquid and micro-particles) [1,7]. In general, their behavior is very complex: interfaces adopt elaborate forms and classification of regimes can sometimes lead to inextricable phase diagrams, where many regimes are mixed up, identified as bubble, slug, or plug, annular, churn, and wispy annular. Several approaches are available for a detection and control in microchannels [8], but, among them all, the optical methods have offered the advantages of a wide range of non-invasive measurement options [9]. The most common optical investigation of microfluidic flows happens by continuous monitoring using a fast Charge-Coupled detector (fast-CCD camera), or a Particle Image Velocimetry (PIV) system [10,11]. Both allow for obtaining detailed and precise flow information with the drawbacks of costly and bulky equipment. The challenge nowadays is to have methodologies based on low cost technologies easily embedded in a portable device for real-time applications. In this context, data-driven approaches based on monitoring optical signals [9] can represent a good alternative since they are non-invasive, offer an easy integration of optical sensors with the microfluidic chips [12,13], and, in future development, the possibility of being even embedded in a chip [14]. In the micro-optofluidic chip presented in [14], the advantage of the integration of micro-optical and microfluics components in one device is proved by taking the advantage of advanced signal analysis methodology to process the optical information and control the flow. Advantages can be also be envisaged for SoC applications due to the simplicity of managing optical signals, as it is proved by a wide literature on flow classification in microchannels [15,16].

In our recent works, advanced signals analysis methodologies have been developed to characterize the flow nonlinearity [17], for data-driven process identification [18,19] and to define parameters for slug flow classification [20] to be used in the development of online control strategy [21]. Starting from the results presented in [20] where the frequency analysis of signals, acquired by a low-cost monitoring setup, was used to classify the air and water dominance inside the micro-channel, in this work, the attention was focused in the development of a methodology suitable for detection of the slug velocity in micro-channels. Additionally, the developed methodology was, in a second step, implemented to realize a platform, easy to be integrated in a SoC, for the real-time slug flow velocity detection.

In the experiments presented in this paper, the slug flow was generated by the interaction of two immiscible fluids {air and water} in two serpentine microchannels of diameter {320 μm and 640 μm}. The serpentine geometry enhancing the unpredictability of the flow guarantees the robustness of the methodologies and the platform presented. The optical-based approach, previously used by the authors, based on the acquisition and processing of optical signals, has been extended to a more general framework. Two methods have been considered: the dual-slit methodology based on the cross-correlation analysis to compute the slug velocity [22] and the spectral analysis to compute the slug frequency [20]. An experimental campaign was carried out by setting nine different flow operative conditions at the inlets. Thanks to the methods used, in each experiment, it was possible to distinguish the water and air slugs velocity and the water and air slugs frequency. The two parameters were also correlated.

The results obtained were used to implement a platform for the real-time detection of the slug velocity. Two experiments were carried out monitoring the slug flow in different operative conditions. The platform performance was successfully validated. The experimental set-up and the analysis approach proposed are presented in Section 1. Then, in Section 2, the results related to data analysis of the experiments carried out are discussed in detail. Finally, in Section 3, the Platform implemented in LabVIEW is described for the real-time detection of the slug velocity showing its performance in two different experiments.

## 2. Materials and Methods

**Figure 1:**
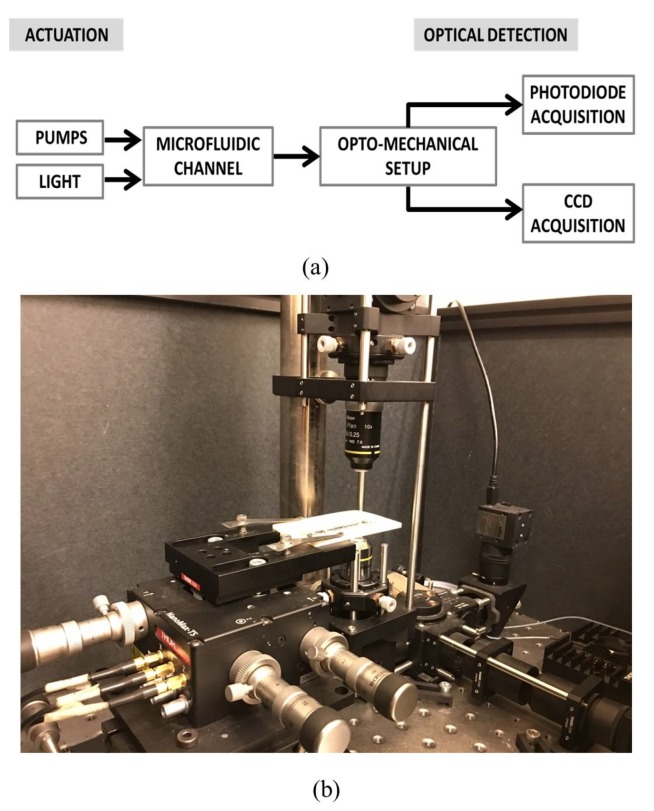
(**a**) The flowchart of the experimental set-up; (**b**) a picture of the opto-mechanical experimental set-up.

**Figure 2:**
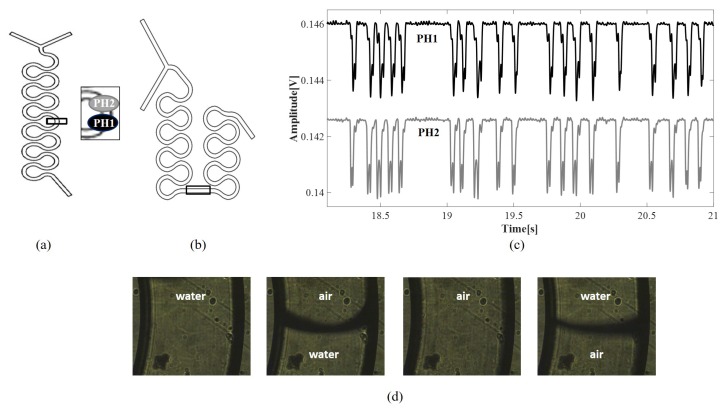
(**a**) the geometry of the serpentine channel of 320 μm (labelled as G-320); (**b**) the geometry of serpentine channel of 640μm (labelled as G-640); (**c**) an example of the trends of the signals acquired by the two photo-diodes {*ph1, ph2*} in G-320 in the experiment with an input flow rate F=0.3 mL/min. The two rectangles in G-320 and G-640 point out the investigation areas where the two-photo-diodes are placed; (**d**) a frame sequence related to the slug passage acquired by the CCD.

A continuous slug flow was generated by pumping de-ionized water and air at the Y-junction of serpentine microchannels in a Cyclic Olefin Copolymer (COC) with a square section and positioned horizontally. Two neMESYS syringe pumps were connected to the two channel inlets. The flow chart and a picture of the complete experimental set-up are shown in Figure 1a,b. The process was monitored in a microchannel area by the simultaneous acquisition of the light intensity variation using a couple of photo-diodes (sample rate of 2 kHz) placed at a distance of 10 mm from each other and a CCD (frame rate of 25 frame/s). The CCD-video was used to have a visual inspection of the process. A frame sequence of the slug passage acquired by the CCD is in Figure 1d. A detailed description of the optical set-up and the signals pre-processing is given in [20]. The serpentine microchannels used in this work are shown in Figure 2a,b, where the two rectangles are placed in the investigation areas. The microchannel in Figure 2a has a diameter of 320 μm and a length of 50 mm (labelled as G-320), and the microchannel in Figure 2b has a diameter of 640 μm and a length of 121 mm (labelled as G-640). The geometry G-320 was used in the experiments carried out to establish an optical signals processing methodology for the computation of slug velocity and slug frequency. Both geometries were considered for the validation of real-time velocity detection by the ad hoc platform implemented. In Figure 2c, an example of the trends of the signals acquired by the couple of photo-diodes {ph1,ph2} in G-320 is shown. As discussed widely in [20], these optical signals are correlated with air/water slugs passage as follows: the top-level represents the water presence in the channel, while the lower level shows the air slugs passage. The two lowest peaks reveal the slugs fronts and rears. Indeed, during the slugs passage, the intensity of the light decreases suddenly due to the difference between the refraction index of COC ($N_{COC}=1.5$) and air ($N_{air}=1$), so the air slug contour becomes darker than the inside of the slug and the chip wall. This effect is less evident during the water passage since the water refraction index (Nwater = 1.3) is closer to the one of the COC. Thanks to this phenomenon, it is possible to clearly distinguish the air and water slugs passage in the signals.

Nine experiments were carried out by feeding equal flow rates of water ($F_{w}$) and air ($F_{a}$) to the two inlets of the microchannel G-320 as in the set F∈{0.1,0.2,0.3,0.4,0.5,0.6,0.7,0.8,0.9}mL/min. The Air Fraction (AF) was respectively AF=0.5. The process was monitored in a position after three bends from the Y-junction; see the insert in Figure 2a. Data were acquired for 20 s, but the first and last 5 s of acquisition were excluded from the analysis.

Three well-known dimensionless parameters widely used in fluid dynamics to establish some basic flow characteristics were computed: the Reynolds number, the Capillary number, and the Dean number [7]. The Reynolds number information is about the transition between laminar and turbulent flow. The boundary value is debatable, but, generally in microfluidics, it is assumed to be Re=1. To enhance the process nonlinearity and be able to evaluate the robustness of the methodology proposed in our experiments, its value was in the range [1,13]. The Capillary number on the order $10^{-3}$, as it is in the experiments presented, assures the slugs’ formation. Finally, the curve in the serpentine geometry can determine a recirculation in the flow in the cross-section. The presence of these phenomena can be evaluated by a Dean number greater than 1, in the experiments ranges in [0.5,6].

These parameters give indication about some characteristics of the flow taken into account: the fluids properties, the microchannel geometry, and input flow rates. Being the process highly nonlinear, it is not sufficient for its dynamical characterization and can not be used for the process control. This underlines the need of data-driven approaches that can monitor and identify the process state in real time.

To establish the velocity of the slug flow in the microchannel, the data were analyzed in time domain by using the dual-slit methodology, presented in [22]. Then, the slug frequency, corresponding to slug passage duration in time, was instigated by spectral analysis using the approach presented by the authors in [20].

### 2.1. The Slug Velocity by Dual-Slit Methodology

**Figure 3:**
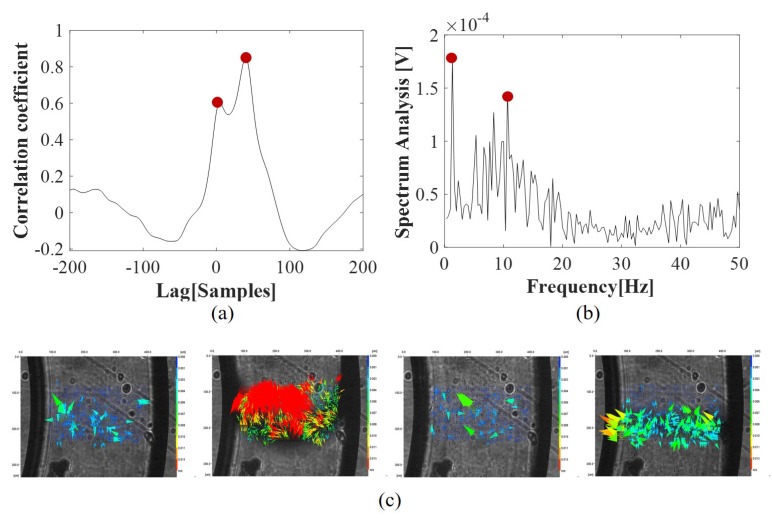
Optical signals processing of {ph1,ph2} in G-320 for the experiment with an input flow rate F=0.3 mL/min. (**a**) an example of the cross-correlation function for the computation of the slug velocity; (**b**) an example of the spectrum of the signal {ph2} for the computation of the slug frequency. In both plots, the two higher peaks are highlighted with red dots; (**c**) a sequence of frames related to a slug passage analyzed by Digital Particle Image Velocimetry (DPIV).

In order to evaluate the slug velocity, the cross-correlation between optical signals acquired by {ph1,ph2} was computed for each experiment. A peak detected in the cross-correlation is representative of the time-delay of the two signals and can be correlated with the time needed by the slug to move from one investigation point {ph2} to another one {ph1}. Knowing the distance between photo-diodes, it is possible to compute the slug velocity using the following formula:

(1) $$v=\frac{d_{s}}{n*T_{s}}$$

where $d_{s}$ is the distance between photo-diodes scaled based on the magnification ($d_{s}=1$ mm), *n* is the delay in samples between the two signals, $T_{s}$ is the sampling period (0.5 ms), and so $n*T_{s}$ is the detectable delay in time [s]. In Figure 3a, the cross-correlation function in a time window of 0.1 s is reported (equivalent to 200samples) for the experiment with F=0.3 mL/min. Two peaks can be detected, one at 2 ms (4samples), and another one at 20 ms (40samples). Based on the signal trend shown in Figure 2c, the first peak can be related to the faster sequence of air slugs that anticipates and follows the passage of a long water slug. The second peak can be related to the passage of long water slugs itself. Detecting different velocities is expectable given the process nonlinearity. Based on the values of these delays, the two velocity values obtained are {0.5 m/s, 0.05 m/s} that refer to the velocity of water and air slugs, respectively.

To validate the results, a sequence of frames obtained related to a slug passage was analyzed by the Digital Particle Image Velocimetry (DPIV) approach, used previously to detect the red blood cell velocity in microchannels in [23]. The mean velocity detected at the slug front and rear was respectively {0.034 m/s, 0.015 m/s}.

### 2.2. The Slug Frequency by Spectral Analysis

The spectral analysis of optical signals for the flow characterization and classification in microchannels was presented in [20] and used in [21] for the real-time slug flow control in a feed-forward configuration. The slug frequency is associated with the duration of slugs passage.

It was implemented by computing the spectrum of the optical signal {ph2}. Then, the spectrum profile was approximated with a multi-mode Gaussian model and the dominant frequencies detected. In both previous works [20,21], the attention was focused on the single-mode Gaussian model and in the highest peak. In this work, two highest peaks were considered in order to investigate the behavior of the water and air slugs. As for the cross-correlation function, the two dominant frequencies were associated with the presence of long water slugs and short air slugs. In Figure 3b, the spectrum of the optical signal {ph2} for the experiment F=0.3 mL/min is shown. The two peaks detected are at frequency f=1.33 Hz and f=10.67 Hz. The highest one can be associated with the passage of long water slugs T=0.75 s, while the other one with the passage of short air slugs T=0.093 s that refer to the frequency of water and air slugs, respectively.

## 3. Velocity and Frequency of Water and Air Slugs

Both methods were used to analyze the optical signals acquired in all the nine experiments F∈{0.1,0.2,0.3,0.4,0.5,0.6,0.7,0.8} mL/min. The signals of the two photo-diodes {ph1,ph2} were used to compute the slug velocity by the dual-slit methodology, whereas in the case of the spectral analysis only the information of one photo-diode {ph2} was used. Finally, the results obtained by the two approaches were mathematically correlated.

**Figure 4:**
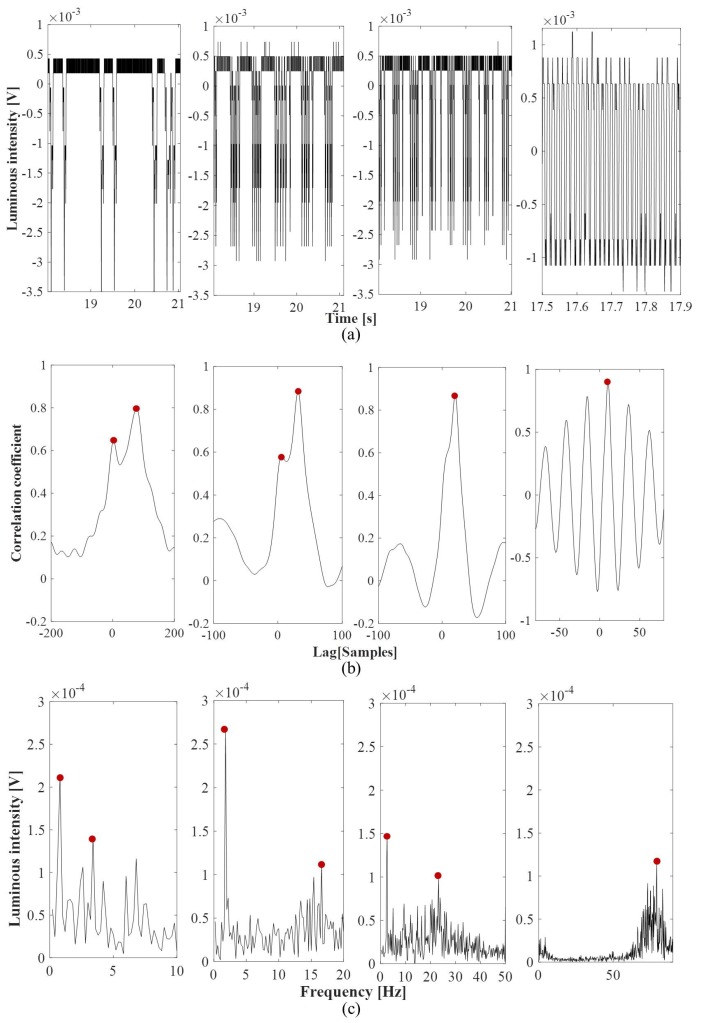
Dynamics of the slug flows in the experiments F∈{0.2,0.4,0.6,0.9} mL/min. (**a**) the trends of the raw optical signals acquired varying the input flow rate. The change of the flow patterns from long water slugs is evident interlaced by short air slugs one after another (F=0.2 mL/min), to longer water slugs (F= 0.4–0.6 mL/min) followed by a train of smaller air/water passages and finally a train of smaller water/air slugs (F=0.9 mL/min); (**b**) the cross-correlation between the signals acquired through the two photo-diodes, and in red dots the peaks detected; (**c**) the spectra of the optical signal {ph2}, and in red dot the peaks detected.

To evidence the signal dynamics in different experimental conditions, in Figure 4a, the trends of the raw optical signals for the experiments F∈{0.2,0.4,0.6,0.9} mL/min are shown. A time window of 2 s was used for F∈{0.2,0.4,0.6} mL/min and 0.5 s for the experiment F=0.9 mL/min. The process nonlinearity does not allow for having a periodic slugs passage; nevertheless, a changing pattern of the slug flows can be clearly distinguished at the increase of the input flow rate, as follows:

- {F= 0.1–0.2 mL/min} long water slugs are interlaced with short air slugs, almost one after another;
- {F= 0.3–0.8 mL/min} long water slugs are followed by a train of short air slugs in sequence;
- {F= 0.9 mL/min} a fast train of short air/water slugs.

In the experiments F∈[0.1,…,0.8] characterized by the passage of long water slugs, a slower dynamic is observed than the one obtained for F=0.9 where an oscillatory-like trend is recognizable.

In Figure 4b, the cross-correlation functions obtained for the four experiments F∈{0.2,0.4,0.6,0.9} mL/min are shown. A time window of 0.1 s (200samples) was used for F=0.2 mL/min and of 0.05 s (100samples) for the other three experiments F∈{0.4,0.6,0.9} mL/min. At the increase of the input flow rate, the convergence of the two peaks in one and a reduction of the time delay can be noticed, with air and water slugs having the same velocity. For the experiment F=0.9 mL/min, an oscillatory trend in the cross-correlation function is evident and a sharp peak at 10samples (5 ms) that stands for a velocity of 0.2 m/s.

The cross-correlation function was computed for all the nine experiments, but two peaks were detected only in the first five F∈{0.1,0.2,0.3,0.4,0.5} mL/min. The method does not seem to be able to differentiate the water and air velocities in the experiments F∈{0.6,0.7,0.8} mL/min, even though we can notice a difference in the flow patterns of Figure 3a. Consequently, it was not possible to have an evaluation of the air velocity only in these conditions. The convergence of the slug flow towards a uniform velocity distribution, having the same water and air slugs velocity, is obtained for F=0.9 mL/min.

**Figure 5:**
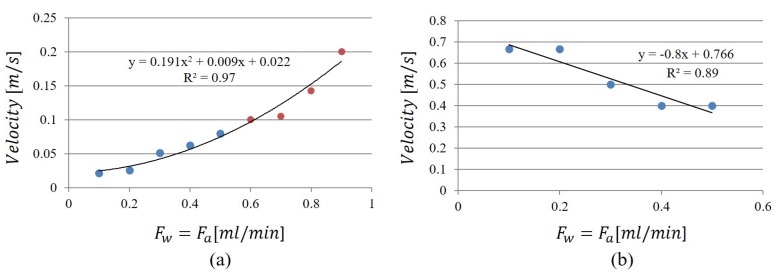
The cross-correlation function was computed for all the nine experiments. (**a**) the values of water velocity obtained by the highest peak were plotted versus the input flow rate: the blue dot are for F∈{0.1,0.2,0.3,0.4,0.5} mL/min and the red dots for F∈{0.6,0.7,0.8,0.9} mL/min; (**b**) the values of air velocity, obtained by the second peak for F∈{0.1,0.2,0.3,0.4,0.5} mL/min.

In Figure 5a, the values of water velocity obtained by the highest peak were plotted versus the input flow rate: the blue dots are for F∈{0.1,0.2,0.3,0.4,0.5} mL/min and the red dots for F∈{0.6,0.7,0.8,0.9} mL/min. In Figure 5b for F∈{0.1,0.2,0.3,0.4,0.5} mL/min, the values of air velocity, obtained by the second peak, were reported. Both graphs were mathematically interpolated. The parabolic increases of the water slug velocity and the linear decreasing of the air slug velocity are worth noticing.

By the analysis of the slug pattern (Figure 4a), the velocity and the frequency of the air and water slugs are expected to be different from each other because of the slower dynamics and the convergence to the same value for {F= 0.9 mL/min}. From this perspective, the analysis in the spectral domain seems more robust.

In Figure 4c, the spectra of the optical signals for the experiments F∈{0.2,0.4,0.6,0.9} mL/min are shown. As expected, at the increase of the input flow rate, the convergence of the two peaks in one and an increase of the frequency of the slugs passage can be noticed, with the air and water slugs assuming the same duration. Coherently with the flow pattern of Figure 3a, one sharp peak is obtained in the experiment F=0.9 mL/min at 79.2Hz that stands for an average duration of the slug passage of 0.012 s.

**Figure 6:**
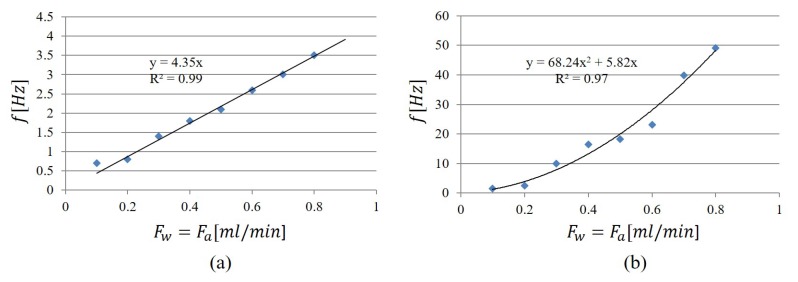
The values of slug frequency computed by the two peaks identified in the spectra versus the input flow rates for the eight experiments F∈{0.1,0.2,0.3,0.4,0.5,0.6,0.7,0.8} mL/min. (**a**) water frequency; (**b**) air frequency.

The values of slug frequency were computed for all the nine experiments and in Figure 6 the values of the two peaks identified in the spectra are plotted versus the input flow rates F∈{0.1,0.2,0.3,0.4,0.5,0.6,0.7,0.8} mL/min. The water frequency is reported in Figure 6a, and the air frequency is reported in Figure 6b. In this case, by the mathematical interpolation at the increase of the input flow rate, the water slug frequency increases linearly in the range [0.5,3.5] Hz, whereas the air slug frequency has a parabolic trend [1,48] Hz. Therefore, a water slug passage can last from [0.3,2] s and an air slug passage [0.02,1] s. The convergence of the two dynamics for F=0.9 mL/min is obtained when the high frequency becomes dominant having the water–air train flow pattern.

Finally, the results of the analysis in the frequency and time domain were graphically correlated. The spectral analysis shows two peaks for all the eight experiments F∈{0.1,0.2,0.3,0.4,0.5,0.6,0.7,0.8} mL/min before reaching the convergence for F=0.9 mL/min. The cross-correlation function has two peaks for five experiments F∈{0.1,0.2,0.3,0.4,0.5} mL/min.

**Figure 7:**
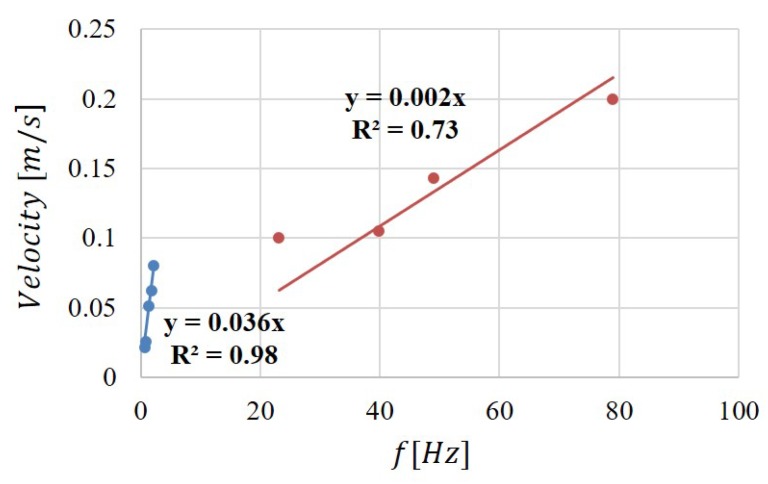
The water velocity plotted versus the water frequency for F∈{0.1,0.2,0.3,0.4,0.5} mL/min (blue dots) and versus the air frequency for F∈{0.6,0.7,0.8,0.9} mL/min (red dots).

In Figure 7, the water velocity was plotted versus the water frequency for F∈{0.1,0.2,0.3,0.4,0.5} mL/min (reported as blue dots) and versus the air frequency for F∈{0.6,0.7,0.8,0.9} mL/min (reported as red dots). The points were linearly interpolated, maintaining the distinction between the input flow ranges. The nonlinearity of the process and its tendency to move from a flow pattern regime to another is underlined by a difference in the two linear interpolations. Additionally, these mathematical relations address the possibility to obtain the dominant velocity of the slug flow through the analysis of one signal in the frequency domain, thus reducing the complexity of the optical set-up and the data analysis.

## 4. Platform for Real-Time Slug Velocity Detection

**Figure 8:**
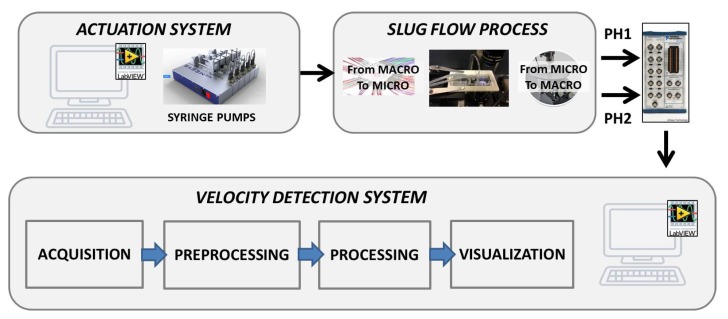
The flowchart of the platform to compute the slug velocity by the analysis of optical signals with the main functional blocks for the data: acquisition, pre-processing, processing, and visualization. The block that manages the pump-computer connection is also pointed out.

After the assessment of the methodology to compute the slug velocity through the analysis of optical signals, a LabVIEW Platform was implemented for a real-time slug flow monitoring. In Figure 8, a flow chart of the system is reported. A module provided by Cetoni (neMESYS SDK software) was integrated in the Platform to drive the syringe pumps. Then, the signals, acquired in cycles based on an established time window, are visualized, pre-processed and processed by using the dual-slit methodology. The velocity values obtained per cycle, for both the air and water slugs, are collected in a chart, showing the trend of the velocity versus cycle. Two experiments were carried out using the microchannels G-320 and G-640 as described in Section 2 and reported in Figure 2a,b.

**Figure 9:**
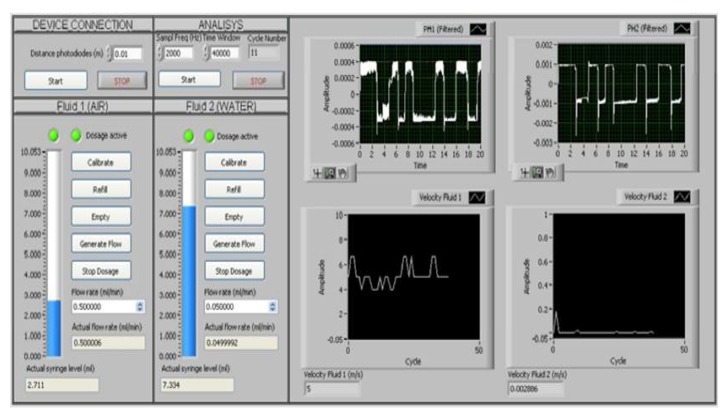
The GUI of the Platform. Two areas are distinguishable: the left area used for user inputting and the right area for data visualization.

In Figure 9, the GUI of the Platform is presented. It is possible to distinguish two areas: the left area, used for inputting the experiment parameters, and the right area, used for the process data visualization.

- In the blank text-boxes at the top of the left area, the user can set the parameters of the experiment: the distance between the photo-diodes, the sample frequency of the acquisition board (in Hz), and the time window length to be analyzed cyclically (in samples). By the text-box in grey, labeled *Cycle Number*, the number of analysis cycles performed is visualized to inform the user about the number of points collected in the velocity chart and the time-horizon monitored. At the bottom of the left area, it is possible to manage the two syringe pumps, set the desired values of the input flow rates (in mL/min), and check the actual values.
- At the top of the right area, the signal acquired by the two pre-processed photo-diodes are plotted in real-time per cycle of 20 s. At the bottom, the charts that collect the water and air velocity are updated cycle-by-cycle. The values obtained for the ongoing cycle are also reported in the text-box.

The implemented algorithm includes five blocks, described in detail below.

- The first block establishes the communication computer–pumps and manages the pumps through some basic procedures as: calibration, refilling, emptying, and start-and-stop of the flow emission. In the GUI of the platform, these block functions are activated by buttons (see Figure 9). The *Start* button in the **DEVICE CONNECTION** section establishes the communication with the pumps, while, in the two **FLUID** sections, we find the buttons to *Calibrate*, *Refill*, *Empty*, *Generate Flow*, and *Stop Dosage* as described previously. There are also two indicators that give feedback regarding the status of the pumps: the first led indicates that the pump is connected, the second one that the dosage is active. The velocity monitoring starts by pressing the *Start* button in the **ANALYSIS** section.
- The second block carries on the two signals acquisition at the established sample frequency and time-window length that subsequently splits them up in two data vectors to be analyzed separately (*Split Signals*).
- The third block (*Filtering*), related to the pre-processing, includes the procedures for: the mean removal, a notch filter (at 50 Hz) and low pass filter. In particular, two first order Butterworth IIR filters with {fc=30 Hz and fc=48 Hz} have been used respectively for *Ph1* and *Ph2*. The system also provides a function for saving the raw and filtered signals.
- The fourth block computes the cross-correlation between the filtered input signals (*Cross-Correlation*). Then, the *Peak Detector* function finds the delay in samples(*n*) related to the two peaks in the cross-correlation and computes the velocities of the two fluids by Equation 1. The system also provides a function for saving those values.
- Finally, the fifth block is for the visualization (*waveform graph*).

### 4.1. Slug Velocity Monitoring in a 320μm Microchannel

The real-time flow information in the serpentine microchannel G-320 was monitored and analyzed by using a sample frequency of 2Khz and in a time-window of 20 s. One inlet of the microchannel was continuously fed with a water flow rate equal to 0.05 mL/min. The air flow rate at the second inlet was varied during the experiment in the set {0.1,0.3,0.5} mL/min, maintaining each value for 13 cycles, and thus was around 260 s. Differently from the experiments discussed in Section 3, the process was slowed-down by decreasing the input flow rate of the water and by setting up an unbalanced configuration of the input flow rate water–air in order to guarantee more stability in the flow velocity [20].

**Figure 10:**
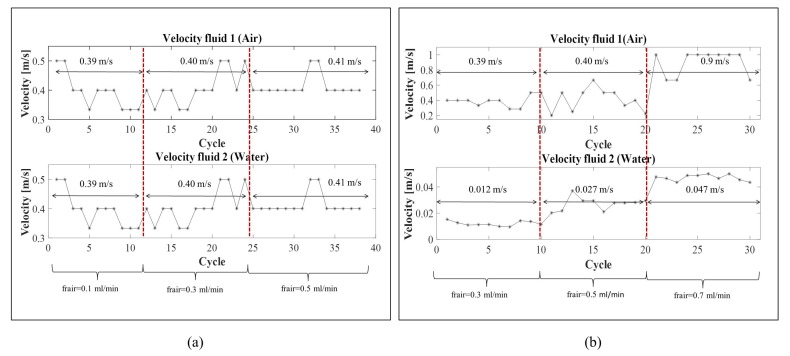
Velocity trends of the water and air fluids computed in real-time (**a**) in the serpentine microchannel G-320 and (**b**) in the serpentine microchannel G-640.

**Figure 11:**
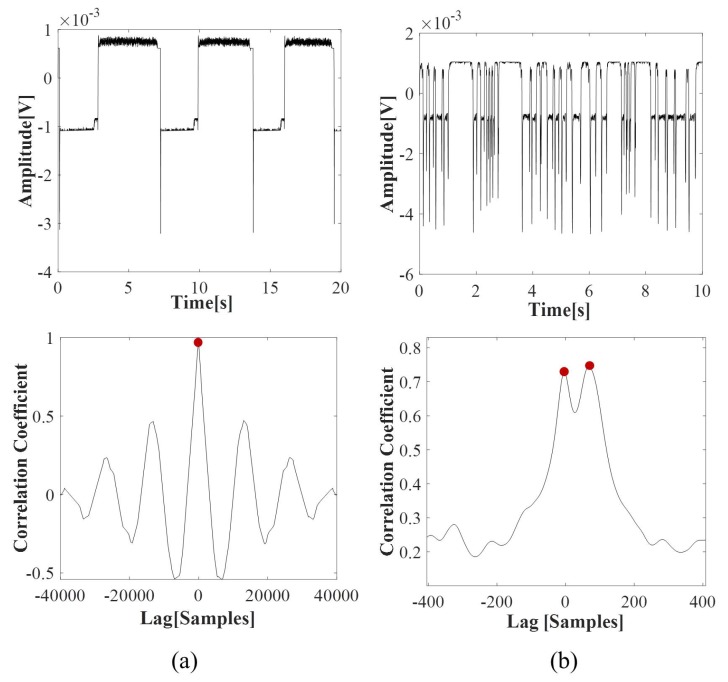
The trends of a signal {ph2} and the cross-correlation functions: (**a**) in the serpentine microchannel G-320 and (**b**) in the serpentine microchannel G-640.

Figure 10a shows the trend of the air (above) and water (below) slugs velocity during the entire duration of the experiment. The red vertical dotted lines separate the three time-intervals in which the air input flow rate was maintained unaltered. The horizontal arrows report the velocity average per interval. As it can be noticed, the air and water slugs velocity was the same, while one peak was identified in the cross-correlation function. Figure 11a shows the trend of a signal {ph2} in the condition air input flow rate 0.5 mL/min and the cross-correlation function obtained. Using very low input flow rates, no great difference is detected in the velocity values in time, but the flow velocity stability increases at the increase of the difference between the two input flow rates, as expected by previous studies [20]. The process nonlinearity leads to the variation of the velocity of 0.1 m/s that is significant considering that the total increase of the velocity is in the range [0.3,0.5] m/s. This phenomenon is particularly evident in the first two input flow rate conditions; then, a stabilization in the process is reached.

### 4.2. Slug Velocity Monitoring in a 640 μm Microchannel

The real-time flow information in the serpentine microchannel G-640 was monitored and analyzed by using a sample frequency of 2Khz and in a time-window of 10 s. The input flow rate of air and water at the two inlets of the microchannel was set equal and varied during the experiment in the set {0.3,0.5,0.7} mL/min, maintaining each value for 10 cycles (around 100 s). A greater diameter leads to a faster transient time, giving the possibility to reach more rapidly the flow steady condition.

Figure 10b shows the trend of the air (above) and water (below) slugs velocity during the entire duration of the experiment. The red vertical dotted lines separate the three time-intervals considered in which the two input flow rates were unchanged. The horizontal arrows report the velocity average per interval. As it can be noticed, the air and water slugs velocity are different. Figure 11b shows the trend of a signal {ph2} in the condition input flow rate 0.5 mL/min and the two peaks in the cross-correlation function. The trends of both air and water slugs velocity increase along the entire experiment. On the other hand, the transient phase is not detectable in the first input condition {0.3} mL/min, which is clearly visible in {0.5,0.7} mL/min.

In this experiment, it is important to notice that the process nonlinearity affects the air slug and water slug velocity differently. The air slug velocity increases in the range [0.2,1.0] m/s; thus, in this case, variations of 0.1 m/s have a minor impact in the velocity detection. As far as the water slug velocity is concerned, they are in the range [0.01,0.05] m/s, but, in this case, the variations detected are very small compared to the air slug velocity. The smoothness in the trend increases underlines the fact that the water flow is less affected by the process nonlinearity than the air flow.

## 5. Conclusions

A challenge in this work was the development of a real-time velocity detection system, for the slug flow analysis in a microchannel based on optical signals monitoring. Due to the nonlinear process, an irregular behavior is expected, so the possibility to use simple low-cost procedures for its monitoring represents an important step in the development of a microfluidic system-on-chip.

In this study, the attention was focused on two-phase flows obtained by the interaction of immiscible fluids {air and water} in two microchannels of 320 μm and 640 μm. The process was monitored through a photo-diode set-up. Two approaches based on the optical signal analysis in time and frequency domain were established and compared. The first one based on the dual-slit methodology was used to establish the water and air slugs velocity. The latter was used to detect water and air slugs frequency that is associated with the slug passage durations. A fulfilling slug flow characterization was obtained in the experimental campaigns by varying the input flow rate. In each experiment, it was possible to distinguish the water and air slugs velocity and frequency. The two parameters were also correlated.

The results obtained were used to implement a platform in LabVIEW for the real-time detection of the slug velocity. Two experiments were carried out monitoring the slug flow in different operative conditions using two serpentine microchannels of diameter {320 μm and 640μm}. The platform performances were successfully validated.

The results obtained represent an important step in the development of non-invasive, low-cost portable systems for micro-flow analysis which could also be suitable for an easy on-chip integration. In future developments, the analysis in frequency domain will be integrated in the platform and its performances will be tested by using different two-phase slug flows.
